# Sex-specific association of high maternal psychological stress during pregnancy on newborn birthweight

**DOI:** 10.1371/journal.pone.0262641

**Published:** 2022-01-20

**Authors:** Nathalie Bernard, Yves Giguère, Joanie Mélançon, Réjean Tessier, George M. Tarabulsy, Jean-Claude Forest

**Affiliations:** 1 Research Center CHU de Québec-Université Laval, Québec, Canada; 2 Department of Molecular Biology, Medical Biochemistry and Pathology, Faculty of Medicine, Université Laval, Québec, Canada; 3 School of Psychology, Université Laval, Québec, Canada; 4 Centre for Research on Youth and Families, Université Laval, Québec, Canada; Medical College of Wisconsin, UNITED STATES

## Abstract

Birthweight is an important predictor of newborn health and has been linked to maternal psychological stress during pregnancy. However, it is unclear whether prenatal stress affects birthweight similarly for both male and female infants. We used a well-established pregnancy cohort to investigate the impact of high maternal psychological stress during pregnancy on birthweight as a function of infant sex. Overall, 5702 mother-newborn pairs were analysed. Of these, 198 mothers reported high levels of stress using the Psychological Stress Measure (nine-items version; PSM-9). Maternal psychological stress was assessed between the 24^th^ and 28^th^ week of gestation and analyses were performed jointly and independently as a function of neonatal sex (separate analyses for male and female infants). Newborns exposed to high maternal psychological stress during pregnancy (a score above 26 measured using the PSM-9 questionnaire, corresponding to >97.5^th^ percentile) were compared to newborns of mothers who reported lower stress. ANCOVAs revealed that high levels of maternal stress during pregnancy were linked to infant birthweight as a function of infant sex. Male infants of mothers who reported high levels of stress had a greater birthweight whereas female infants had a lower birthweight under the same conditions, in comparison to mothers who did not report greater levels of stress. Although the effect size is small, these results underline the possibility that male and female fetuses may use different strategies when adapting to maternal adversity and highlight the need to consider infant sex as a moderator of the association between maternal psychological stress during pregnancy and infant birthweight.

## Introduction

Birthweight is an important marker of infant health [1]. Low-birth weight (LBW, <2500 g) and macrosomia (>4000 g) increase the risk of morbidity and mortality [2, 3]. LBW babies are at greater risk for chronic lung disease, cerebral palsy, deafness, epilepsy, learning disabilities and attention deficit disorders [4–6], while macrosomic babies are at greater risk for shoulder dystocia, clavicular fracture, instrumented vaginal delivery, emergency caesarean section and neonatal hypoglycemia [7–9]. Birthweight has been linked to different pregnancy-related variables, such as excessive maternal weight gain during pregnancy and gestational diabetes (GDM) both of which trigger increased fetal growth [8]. Also, pre-existing maternal conditions, such as a pre-pregnancy body mass index (BMI) >30, parity >4 and advanced age (>40) contribute to higher infant birthweight [8, 10, 11]. In contrast, LBW is more common in infants from low socioeconomic backgrounds, as well as when mothers present chronic hypertension or nephropathy, or tobacco, alcohol or drug consumption [12]. Hypertensive disorders of pregnancy (HDP) and in particular preeclampsia (PE), have been associated with adverse birth outcomes, including LBW and small for gestational age (SGA) [13, 14].

In recent years, maternal psychological stress (MPS) during pregnancy has also been proposed to play a role in the control of birthweight [15–18]. Different studies have shown that MPS during pregnancy is inversely associated with birthweight and gestational age at delivery [16]. Hypothalamic–pituitary–adrenal (HPA)-axis activity has been postulated to play a mediating role in the relationship between maternal psychological stress and birth outcomes, such as birthweight [15, 19, 20]. Exposure to stress activates the HPA axis resulting in the release of multiple hormones, including cortisol, for expecting women [21, 22]. Increased maternal cortisol secretion may lead to the increased secretion of placental corticotropin-releasing hormone (CRH), resulting in decreased fetal growth and size at birth [15, 23].

There are known differences in birthweight between boys and girls, with boys being heavier than girls [24]. It has been proposed that sexually dimorphic differences in growth are mediated by the sex-specific function of the placenta, since it is an important mediator for fetal development and growth [24]. In a 2010 review, Clifton hypothesized that male and female fetuses use different strategies to adapt to similar maternal adverse conditions. Male fetuses are hypothesized to institute minimal placental adjustments, all of which would target normal growth, placing them at greater risk of adverse outcome in the presence of a stressful event [24, 25]. Inversely, female fetuses respond to intrauterine adversity through multiple adjustments in the expression of placental genes and proteins, minimally reducing their growth, and allowing them to continuously adjust to other challenges that may emerge in the intrauterine environment as the pregnancy unfolds [24, 25].

Furthermore, there may be sex-specific differences in fetal steroid production from the human fetal-placental unit that may influence placental function [24]. Placentas from female fetuses were shown to be responsive to changes in glucocorticoid concentrations, whereas placentas from male fetuses appear resistant to such changes. Such differences suggest that fetal growth may not be similarly stimulated with greater levels of maternal prenatal cortisol secretion [24, 26].

In support of this possibility, some studies have obtained results that suggest that fetal adaptation to stress in the prenatal environment may vary according to fetal sex [25–30]. Kaitz *et al*. found that moderate levels of anxiety (measured with the Beck Anxiety Inventory) may affect birthweight differently for males and females [25, 29]. They observed that male neonates of mothers experiencing greater levels of anxiety weighed more than those of mothers who did not experience greater anxiety, whereas female neonates weighed less under the same circumstances [25, 29]. Relatedly, Togher et al. reported that exposure to second trimester maternal anxiety negatively affected female birthweight, but not male birthweight [26].

Pregnancy is linked to many physical and emotional changes, which may affect maternal experience of prenatal stress. At moderate levels, stress is considered as a normal and even useful state, allowing individuals to adapt to different circumstances and challenges [31, 32]. However, high levels of psychological stress during pregnancy, due in part to the release of high levels of cortisol, may be harmful to both maternal and infant health and developmental outcome [32–34]. One of the mechanisms by which stress may be linked to infant outcome may be via an association with birthweight, an association that may vary as a function of infant sex.

The purpose of this study is to examine the hypothesis that high levels of MPS are linked to neonatal birthweight. The potential moderating effect of newborn sex is also examined. MPS was assessed at the end of the second trimester between the 24^th^ and 28^th^ week of gestation. The potential contributions of socioeconomic and obstetrical variables as potential confounding factors were controlled for in this well-establish pregnancy cohort study.

## Materials and methods

### Study design and data collection

**Fig 1:**
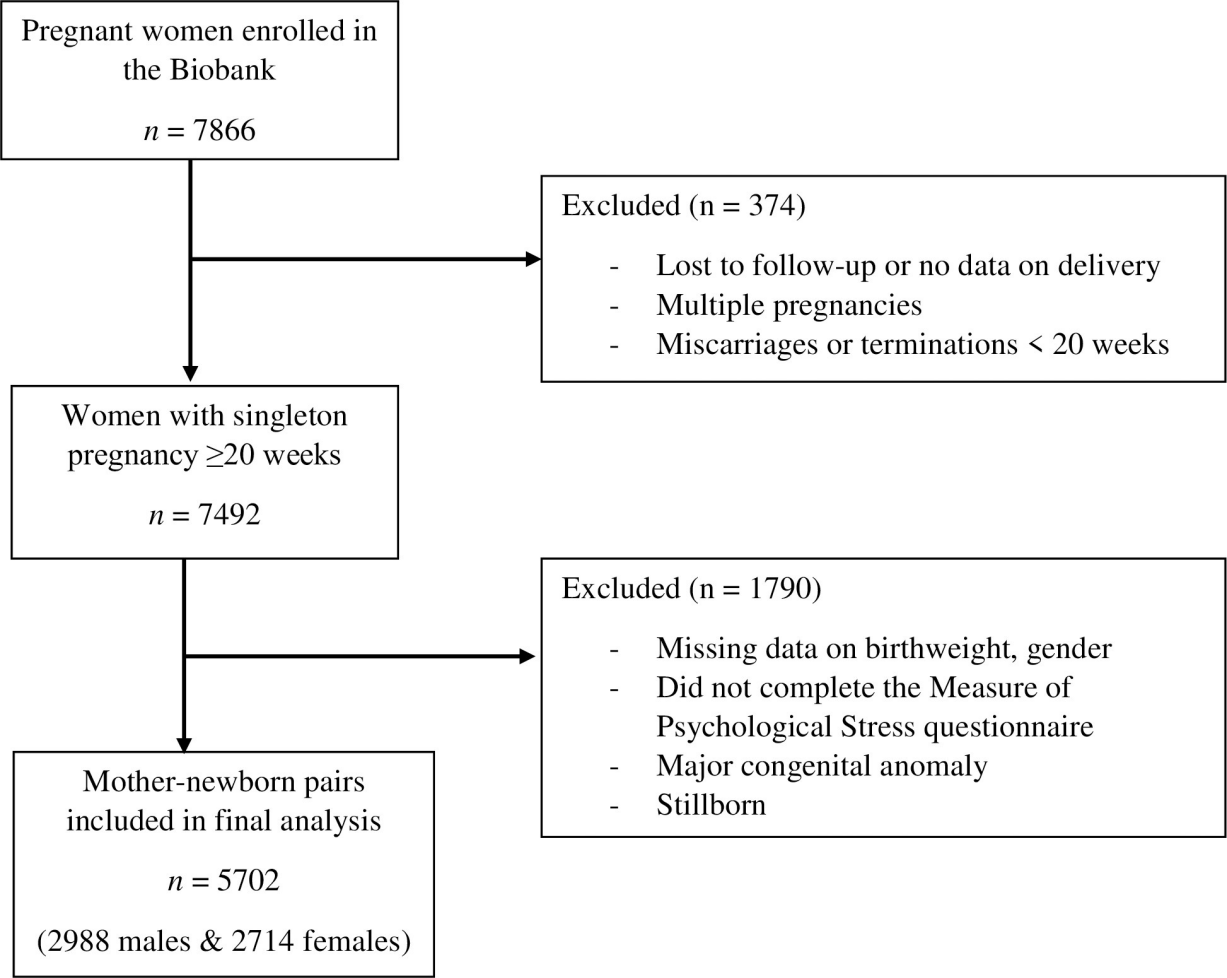
Flow chart of the sample selection.

This study is based on a large prospective cohort of 7866 pregnant women recruited at the CHU de Québec-Université Laval from April 2005 to March 2010 during their first prenatal visit (median 15 weeks) to the perinatal clinic of the institution. Details of the original study design may be found elsewhere [35–38]. Pregnant women aged 18 years or older (mean 29.3; range 18.1–44.8) without chronic hepatic or renal disease were eligible to participate in the study. Exclusion criteria for the present study included women lost to follow-up, multiple pregnancies, and pregnancy terminations (voluntary or medical interruption of pregnancy (VIP or MIP)), miscarriages or fetal deaths before 20 weeks of gestation. This left a sample of 7492 women with a singleton pregnancy of more than 20 weeks. From this sample, 1790 women were excluded because the measure of stress was not completed, there was other missing data, or the child was born with major congenital anomaly or was stillborn. The babies with a birthweight below 1500 g (considered VLBW) were excluded from analyses (9 females, 6 males). The final sample included 5702 mother-newborn pairs, with 2988 male and 2714 female newborns (Fig 1).

Documentation on pregnancy and delivery was obtained following delivery via a standardized prenatal follow-up form (gynecological and obstetrical history, presence of illness or disorders, medications used, etc) completed at each prenatal visit by the nurse and the physician and included in patient charts. Between the 24^th^ and the 28^th^ week of gestation, women were invited to complete a questionnaire (in French) that included information about their past medical and family history, habits (e.g., use of tobacco, alcohol, drugs, frequency of physical activity, nutritional habits, etc.), sociodemographic characteristics (ethnicity, marital status, annual household income, highest level of education, employment status, etc.), anthropometric measures (age, height, weight), and perceived level of stress. Participants gave written informed consent and the study was approved by the CHU de Québec-Université Laval Ethics Review Board (initial approval date: 9 November 2004, Project 5-04-10-01 [95.05.17l SC12-01-159).

### Psychological Stress Measure (PSM)

The Psychological Stress Measure (nine-item version) was used to collect data on maternal perceptions of stress. The PSM is a validated questionnaire used to assess stress symptoms felt by someone in the last four to five days. It is intended to be an indicator of the current experience of stress, as opposed to a marker of symptoms of depression or anxiety [39]. In recent years, the PSM has been used in different study contexts [40–45]. Developed by Lemyre and Tessier, this unidimensional scale addresses three distinctive domains of stress: cognitive-affective, somatic, and behavioral [39, 46, 47]. The original version of this scale contains 45 items. A shorter nine-item version was created in 2002 to meet research needs. This version meets the same reliability and validity criteria as the original measure, has an internal consistency of 0.89, and also contains the three stress domains that were part of the original scale [39, 46, 47]. A four-point likert scale is used to answer each of the nine stress items: (1) “Never”, (2) “Sometimes”, (3) “Often”, (4) “Very Often”. The cut-off points were established from two large-scale studies in general populations. Scores ranging between 9 and 15 are classified as low stress (perceived stress ≤ the population average), between 16 and 25 as intermediate stress, and above 26 as high stress (>97.5^th^ percentile). For the purpose of the present analyses, women were divided into two groups: women exposed to high stress (>97.5^th^ percentile) and women exposed to low or intermediate stress (reference group).

Validity with expecting mothers is currently being gathered. In a study involving 143 pregnant mothers, we have found PSM scores to be linked to those from the Symptom Checklist (SCL-90-Revised) when measures were taken at the same time (*r* = 0.59; p< .0001). Furthermore, in the present study, the PSM is strongly correlated with the short version of the Ilfeld Psychiatric Symptom Index (*r* >0.75; Cronbach alpha coefficients of .80 and .86), a measure widely used in large epidemiological surveys in Quebec and validated by way of a large sample representative of the Québec population aged over 18, men and women [48, 49].

### Statistical analyses

Analyses were performed in combination (male and female neonates together) where direct effects were considered as moderation by neonate sex. Analyses were also performed independently as a function of neonate sex (separate analyses for male and female infants). Characteristics of mothers exposed to high stress and those in the reference group were compared using the z-test or Fisher’s exact test for categorical variables and the Mann-Whitney U-test for continuous variables. Continuous variables are expressed as mean ± one standard deviation (SD). Since we were interested by the impact of a high level of stress (>97.5^th^ percentile) on the birthweight of male and female newborns, we performed a group-based analysis comparing newborn birthweight as a function of PSM level (high level of stress vs. reference stress group).

Factors that were considered as potential confounders of the association between perceived stress and birthweight were selected based on the current literature and biological plausibility, and included: maternal age, parity, pre-pregnancy body mass index (BMI), ethnicity, smoking status, alcohol and drug use during pregnancy, antidepressant or anxiolytic use during pregnancy, presence of HDP or GDM, weight gain during pregnancy, annual household income, highest level of education, marital status and gestational age at delivery. After testing, the pattern of missingness was assumed to be missing at random (MAR). For continuous variables, missing values were estimated by the multiple imputation algorithm, (using the Markov Chain Monte Carlo method with 100 imputations). For categorical variables, the missing values were replaced by a missing indicator variable (“unknown”). There were no missing data in the following potential explicative variables: maternal age, gestational age at delivery, parity, presence of gestational diabetes mellitus (GDM) and hypertensive disorders of pregnancy (HDP), and birthweight. Less than 5% of the data were missing for the other covariates, except for annual household income (7.23%) and for weight gain during pregnancy (in boys: 13.29%; in girls: 11.53%).

Unadjusted analyses were performed by Analysis of variance (ANOVA) while Analysis of covariance (ANCOVA) were used to evaluate whether birthweight means (dependent variable) are equal across groups of maternal psychological stress during pregnancy (independent variable). We used a backward model and started by simultaneously adding all confounders listed above. P-values of < .05 were needed for any variable to be retained in the multivariate model. Since birthweight is strongly related to gestational age, we also performed the analyses after excluding preterm (<37 weeks of gestation) newborns. We also analyzed the association of newborn sex using standardized sex-specific birthweight Z-scores for gestational age as a dependent variable. Statistical analyses were performed using XLSTAT (2020.1.1 version, Addinsoft).

## Results

In the study cohort, 42.4%, 54.1% and 3.6% of women reported respectively low, intermediate and high levels of stress during pregnancy. First, the concomitant effect of PSM score and sex of the newborn on the birthweight was evaluated by ANOVA. As expected, male newborns had significantly higher birthweight than female newborns, in both stress groups. However, comparing by pairs (with a 95% CI (CI_95%_)) showed that high levels of maternal psychological stress accentuated this difference. Indeed, female newborns of mothers exposed to high psychological stress had significantly lower birthweight than female newborns of mothers not exposed to high stress (Difference: -136.74 g CI_95%_ [-12.73 - -260.75]; p = .004). In male newborns, there was a trend for greater birthweight in newborns of mothers exposed to high PSM (Difference: 102.98 g CI_95%_ [-53.65–259.61]; p = .083). The interaction between stress level and newborn sex was statistically significant, explaining 0.2% of birthweight variance (F = 11.976; ƞ^2^ = 0.002; p = .001).

Table 1 summarizes the characteristics of the mothers included in this study according to the sex of their newborn and their level of stress. Among males, 2.91% experienced high maternal psychological stress during the second trimester, compared to 4.09% in females. These rates were significantly different.

**Table 1:** Characteristics of mothers as a function of newborn sex and stress level.

	Males (n = 2988)		Females (n = 2714)	
	Level of stress		Level of stress	
	Reference group	High	Reference group	High
	(n = 2901)	(n = 87)	(n = 2603)	(n = 111)
**Maternal age (years)** ^**a**^	29.57 ± 4.26	28.76 ± 4.26	29.58 ± 4.23	29.23 ± 5.45
**% Nulliparous**	47.88%	39.08%	47.18%	42.34%
**% White**	96.99%	93.83%	97.24%	95.24%
**% High school diploma or less**	26.47%	33.72%	27.72%	35.45%
**% Annual household income <40 000$**	20.90%	27.71%	21.53%	39.80%^b^
**% Marital status (single)**	6.67%	18.39%^b^	6.82%	10.81%
**% Smokers during pregnancy**	11.88%	25.29%^b^	11.99%	22.52%^b^
**% Alcohol during pregnancy (>1/week)**	0.94%	2.38%	1.05%	0.96%
**% Drugs during pregnancy (yes)**	2.59%	1.19%	2.01%	3.64%
**Pre-pregnancy BMI (kg/m** ^**2**^ **)** ^**a**^	24.20 ± 5.21	24.98 ± 5.97	24.14 ± 5.11	24.88 ± 5.86
**Gestational age at delivery (weeks)** ^**a**^	39.37 ± 1.42	39.32 ± 1.75	39.46 ± 1.34	39.24 ± 1.41^b^
**Weight gain during pregnancy (kg)** ^**a**^	15.05 ± 5.40	16.54 ± 7.04	14.64 ± 5.45	14.13 ± 5.78
**% GDM**	7.76%	10.34%	6.15%	6.31%
**% HDP**	5.21%	1.15%	4.34%	8.11%
**PSM score** ^**a**^	16.48 ± 3.41	28.13 ± 2.37^b^	16.28 ± 3.41	27.88 ± 1.96^b^
**Birthweight (g)** ^**a**^	3486 ± 483	3590 ± 550^b^	3378 ± 464	3241 ± 488^b^

^a^mean ± SD

^b^p< .05

BMI: body mass index; GDM: gestational diabetes mellitus; HDP: hypertensive disorders of pregnancy; PSM: Psychological stress measure

As shown in Table 2, birthweight was strongly correlated with gestational age at delivery.

**Table 2:** Pearson correlations between covariables included in model.

	High stress	Birthweight
Maternal age	-0.024	0.028^a^
Gestational age at delivery	-0.018	0.498^a^
Weight gain during pregnancy	0.011	0.204^a^
Pre-pregnancy BMI	0.027^a^	0.160^a^
HDP	0.002	-0.080^a^
GDM	0.008	0.003
Nulliparous	-0.024	-0.137^a^
Smoker	0.066^a^	-0.129^a^
Female newborn	0.032^a^	-0.122^a^
Male newborn	-0.032^a^	0.122^a^
High stress	**1**	-0.016
Birthweight	-0.016	**1**

BMI: body mass index; HDP: hypertensive disorders of pregnancy.

GDM: gestational diabetes mellitus

^a^p< .05.

Using a backward model, starting by simultaneously adding all potential confounders, nine covariates contributed to birthweight and were included in the multivariate adjusted model. None of the major socio-economic covariates (education, marital status, annual household income, drugs and alcohol uses) were retained in the backward models. No multicolinearity between covariables was observed in the multivariate model (TOL>0.8 and VIF<2). In the combined model (males + females), 37% of birthweight variance was explained by the variables in the model (maternal age, pre-pregnancy BMI, weight gain during pregnancy, parity, presence of GDM and HDP, gestational age at delivery, smoking status, level of stress, sex of newborn, and the stress by newborn sex interaction). The stress by newborn interaction was statistically significant, accounting for 0.1% of birthweight variance (ƞ^2^ = 0.001; p = .003).

Table 3 shows the variables included in the final model. The effect size of the stress by newborn sex interaction was small, but significant. Among the explanatory variables, as was expected, gestational age accounted for most of the variance.

**Table 3:** Analysis of the covariance in the final model.

	DF	Sum of Squares	F	ƞ^2^
Model	13	488074773.58	259.72^a^	0.37249
Maternal age	1	1625935.06	11.25^a^	0.00124
Gestational age at delivery	1	285007518.35	1971.60^a^	0.21751
Weight gain during pregnancy	1	45151433.99	312.35^a^	0.03446
Pre-pregnancy BMI	1	45806926.94	316.88^a^	0.03496
Level of stress	1	14374.59	0.10	0.00001
HDP	1	2830348.92	19.58^a^	0.00216
GDM	1	1164023.89	8.05^a^	0.00089
Parity	1	38387126.51	265.55^a^	0.02930
Smoking status	3	21999234.88	50.73^a^	0.01679
Sex of the newborn	1	7610129.77	52.65^a^	0.00581
Level of stress*sex of the newborn interaction	1	1302534.95	9.01^a^	0.00099

DF: degrees of freedom; BMI: body mass index; HDP: hypertensive disorders of pregnancy; GDM: gestational diabetes mellitus

^a^p< .05.

**Fig 2:**
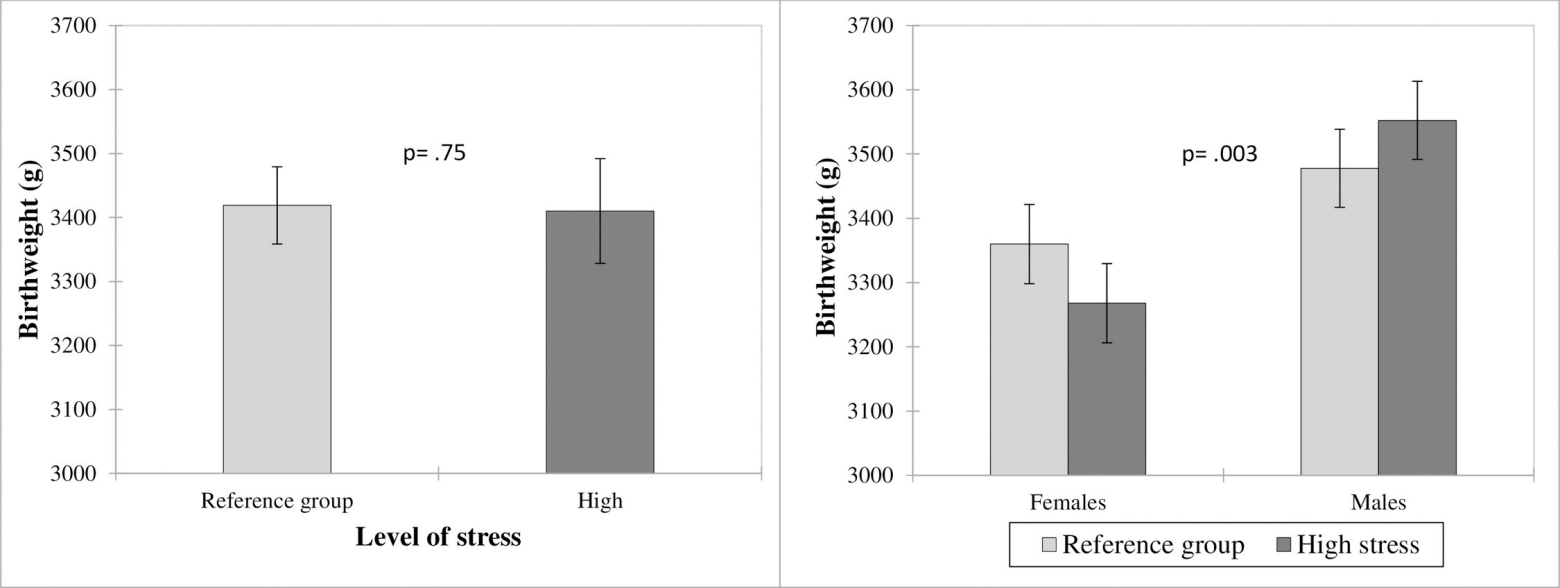
Neonate birthweight as a function of maternal psychological stress during pregnancy and newborn sex. Results presented as Least Square (LS) means with CI_95%_; variables included in the model: maternal age, pre-pregnancy BMI, weight gain during pregnancy, parity, presence of GDM and HDP, gestational age at delivery, smoking status, level of stress, sex of the newborn and ‘level of stress*sex of the newborn’ interaction.

This interaction is broken down in Fig 2 which compares birthweight as a function of maternal psychological stress during pregnancy, overall and as a function of newborn sex (Fig 2). In comparison to infants of women not exposed to high stress (reference group), neonates whose mothers experienced high PSM did not vary in their birthweight (Difference: -8.77 g). Analyses based on neonate sex revealed significant differences in birthweight. Male newborns whose mother experienced high levels of PSM trended to have greater birthweight (Difference: 74.50 g CI_95%_ [-12.06–161.06]; p = .09), whereas female newborns in the same circumstances had significantly lower birthweight (Difference: -92.03 g CI_95%_ [-18.63 –-163.44]; p = .014).

To better evaluate the link between PSM and birthweight as a function of newborn sex, we also performed analyses independently for male and female infants. The results supported those seen in combined model (males + females). Very high PSM (>97.5^th^ percentile) was marginally linked to increased birthweight by 80.63 g CI_95%_ [-6.44–167.70] in males, while conversely greater levels of PSM was linked to significantly lower birthweight by -91.17 g CI_95%_ [-17.60 - -164.73] (p = .015) in females. PSM effect size was small, but significant in female newborns (F = 6.38; ƞ^2^ = 0.001; p = .012) (see S1 File).

**Fig 3:**
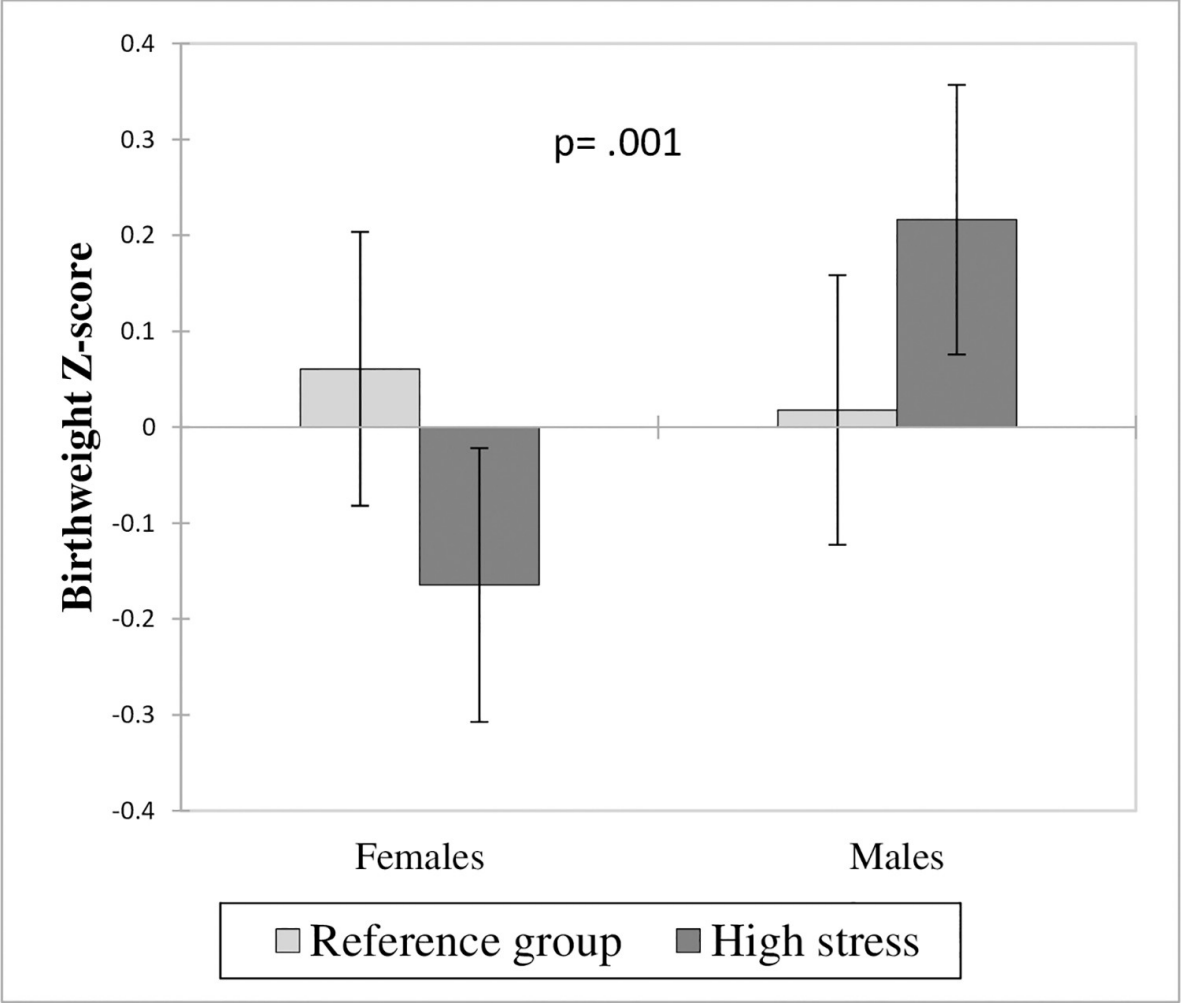
Neonate birthweight Z-score as a function of sex of the newborn according to maternal psychological stress during pregnancy. Results presented as Least Square (LS) means with CI_95%_; variables included in the model: maternal age, pre-pregnancy BMI, weight gain during pregnancy, parity, presence of GDM and HDP, gestational age at delivery, smoking status, level of stress, sex of the newborn, and ‘level of stress*sex of the newborn’ interaction.

To further measure whether female and male neonates differ within levels of stress, we used standardized sex-specific birthweight Z-scores for gestational age in analysis. Birthweight Z-scores were calculated using reference curves derived from population-based Canadian references [50]. Fig 3 shows the neonate birthweight Z-score as a function of newborn sex and maternal psychological stress levels during pregnancy. After adjusting for covariates, results reveal that male and female newborns in the reference group have a similar birthweight Z-score (Difference: 0.043; p = .065), whereas newborns of mothers exposed to high stress are different: birthweight Z-score was significantly lower for females and greater for males (Fig 3) (Difference in males: 0.198 CI_95%_ [0.005–0.391], p = .044; Difference in females: -0.225 CI_95%_ [-0.054 –-0.397], p = .01). ANCOVA results using birthweight Z-scores as the dependent variable showed an effect size of the stress by sex interaction greater than the effect size observed using birthweight as dependent variable (ƞ^2^ = 0.002, F = 11.309, p = .001), but remained small. Once again, analyses performed independently for male and female infants supported the observed results in the combined model (see S2 File).

Since birthweight was strongly related to gestational age, we also performed the analysis after excluding preterm births. There were no births prior to 30 weeks of gestation. 294 infants (5.16%) were born prior to 37 weeks (167 in males, 127 in females). When analyses were conducted solely with full-term births (≥37 wks), results were unchanged in both combined model and analyzes performed by sex neonates (see S3 File).

## Discussion

In this study, we tested the hypothesis that the birthweight of newborns whose mothers experienced high levels of maternal psychological stress during pregnancy varied as a function of newborn sex in comparison to that of newborns whose mothers who did not report a high level of stress. The present study revealed that neonatal birthweight varied as a function of PSM during pregnancy. This variation in PSM, measured during the second trimester of pregnancy, is sex specific: when mothers reported very high levels of stress, birthweight was greater when neonates were male and lower when they were female, in comparison to neonates of mothers who did not reported high levels of stress. Adjustment for potential confounding variables did not change these observations, suggesting that male and female fetuses respond differently to MPS, which should be considered as a potential determinant of birthweight. However, it is important to underline that the relative effect size for PSM was small and not yet applicable in clinical settings. Unsurprisingly, gestational age had the greatest effect on birthweight among the explanatory variables.

Although the effect size is small, it is relevant from a pathophysiological and an epidemiological point of view. Our results support those of smaller studies showing sex differences in fetal response to MPS and anxiety. Kaitz *et al*. found that male and female fetuses respond differently to maternal gestational anxiety measured in the third trimester, where accelerated growth of male fetuses (n = 38) and reduced growth of female fetuses (n = 70) was noted in the context of maternal prenatal anxiety [25]. In a cohort of 55 newborns (25 males, 30 females), Togher *et al*. observed a link between maternal anxiety in the second trimester and lower birthweight in female but not male infants [26]. Rosa *et al*. found that prenatal stress was not associated with birthweight z-scores (n = 527) but observed that increased prenatal stress was linked to shortened gestational age at delivery and PTB risk in male infants [30]. Our results based on a large cohort replicate and confirm that the effect of high maternal psychological stress during pregnancy on birthweight generates sex-specific responses.

These findings suggest that male and female fetuses use different mechanisms to cope with adverse intrauterine environments such as those that might emerge from MPS during pregnancy. There is increasing evidence that suggests that male fetuses continue or accelerate their growth in the face of challenge, and in contrast, female fetuses engage in a minor reduction of their growth [24, 25]. A possible mechanism accounting for such divergent fetal responses involves placental reaction to maternal HPA-axis functioning under stress. When the expecting women experience stress, the HPA-axis is activated and multiple hormones, such as cortisol, are released [21, 22]. In a review by Clifton, it was suggested that birthweight differences may be attributed to variations in placental pathways associated with the operation of such hormones. The placenta of female fetuses adjusts its glucocorticoid metabolic activity in the presence of high maternal glucocorticoid concentrations. In the male fetus, the stress response activates testosterone dependent pathways which may inhibit glucocorticoid regulated pathways [24]. Eriksson *et al*. suggested that males’ placentas may be more efficient at extracting nutrients than females’ placentas but may have less reserve capacity to store energy [51]. Present results lend credence to this hypothesis and suggests that further study be made of such processes. Moreover, Tamini *et al*. observed that the energy intake of pregnant women is about 10% higher when they are carrying a male rather than a female fetus, supporting the hypothesis that women expecting male infants may have higher energy requirements [52]. Interestingly, among males, 2.91% experienced high maternal prenatal psychological stress during the second trimester, compared to 4.09% in females. Our results support those of a recent study by Walsh *et al*. who observed that maternal prenatal stress during pregnancy influenced offspring neurodevelopment and birth outcomes including the ratio of male to female newborns, showing reduced male births in contexts where mothers experienced high levels of stress [53].

Our study has some limitations. The prevalence of high maternal psychological stress during second trimester was low (3.47%) and it is not clear when in the process MPS may impact obstetrical outcomes. Certainly, different researchers have argued in favor of the second trimester being a time when the fetus is more vulnerable to MPS, but results in this regard have been mixed [54]. Moreover, although we have adjusted results for many confounding variables, we cannot exclude the possibility that other unidentified confounding variables may account for part of the observed associations. One such variable concerns the duration of the experience of stress. The questionnaire that was administered asked women to report on their experience of stress during the four to five days before completing the measure. Mothers reported on their experience of stress only once during their pregnancy, between the 24^th^ and 28^th^ week of gestation. The origins of their stress experience remains unknown. It was not possible to know whether their experience referred to circumstances, emotions and cognitions that took place during their pregnancy or that predated it. Since some pathophysiological modifications at the placental level begin during the first trimester, we cannot exclude the possibility that the observed effect reflect the presence of stress in the first or second trimester. Further study involving repeated measures of stress are required to more accurately tease out the effect of MPS in this regard. It is noteworthy that recent studies found that perceptions of stress measured during the three trimesters of pregnancy are stable [55, 56], and that measures of prenatal stress are related to maternal experiences of postnatal stress, as we have recently observed, suggesting the presence of stability in maternal reports of stress during the perinatal period [57]. The effect size is small and not yet applicable in clinical settings. Our results must therefore be interpreted with caution.

However, this study also presents certain strengths. This is the largest study to have addressed this issue, involving 5702 mother-newborn dyads, including 198 fetuses exposed to very high levels of MPS during the second trimester. The high number of mother-newborn dyads allowed us to maintain sufficient statistical power after stratification by neonate sex to test for the stress by newborn sex interaction Our analyses underlined a sex-specific effect of MPS on birthweight. Moreover, the homogeneity of participants in this study is another favorable characteristic, with predominantly women involved in a public health system where all pregnant women have access to similar pregnancy monitoring. While it is important to conduct this study in situations where national and ethnic factors are considered more thoroughly, the homogeneity of the participants in this study reduces the probability that specific characteristics of the sample are responsible for results.

## Conclusion

Although the effect size is small, high levels of MPS during the second trimester is associated with birthweight in a sex-specific manner. In both groups (reference and high stress groups), male newborns had a significantly greater birthweight than female newborns. Interestingly, a high level of stress accentuated this difference. Male neonates of mothers exposed to high levels of stress had a greater birthweight compared to neonates of mothers not exposed to high stress. In contrast, in female babies, high MPS was significantly linked to lower birthweight. Our results may generate new research hypothesis, which may provide a better understanding of the effects of stress on expecting mothers relative to the sex of the fetus. For example, our results underline the possibility that male and female fetuses respond differently to intrauterine challenges, such as those posed by MPS and highlight the need to consider other potential determinants of birthweight.

## Supporting information

S1 File Neonate birthweight as a function of maternal psychological stress during pregnancy: Analyzes by sex of the neonate. (PDF)

S2 File Neonate birthweight Z-score as a function of maternal psychological stress during pregnancy: Analyses by sex of the neonate. (PDF)

S3 File Term neonate birthweight as a function of maternal psychological stress during pregnancy. (PDF)
