# Supplementary material for: Sex-specific association of high maternal psychological stress during pregnancy on newborn birthweight
Source: PLoS One. 2022 Jan 20;17(1):e0262641. doi: 10.1371/journal.pone.0262641 (PMC8775189; doi:10.1371/journal.pone.0262641)
Supplement: S1 File — (PDF) [file pone.0262641.s001.pdf]

Neonate birthweight as a function of maternal psychological stress during pregnancy: Analyses by sex of the neonate

To better evaluate the link between PSM and birthweight as a function of newborn sex, we also performed analyses stratified by sex of infants. After adjustment for maternal age, pre-pregnancy BMI, weight gain during pregnancy, parity, presence of GDM and HDP, gestational age at delivery and smoking status, very high PSM (>97.5th percentile) was marginally linked to increased birthweight by 80.63 g ($CI_{95\%}$ [-6.44 – 167.70]; standardized coefficient β= 0.028 $CI_{95\%}$[-0.002 – 0.058]; p= .070) in males. Conversely, greater levels of PSM was linked to lower birthweight by 91.17 g in females ($CI_{95\%}$ [-17.60 – -164.73]; standardized coefficient β= -0.039 $CI_{95\%}$[-0.070 – -0.007]; p= .015) in a model adjusted for pre-pregnancy BMI, weight gain during pregnancy, parity, presence of HDP, gestational age at delivery and smoking status. In each model, 36% of birthweight variance was accounted for (S1 Table). The information provided by the explanatory variables was significantly better compared to what would explain the sole mean of the birthweight. Among the explanatory variables, as was expected, gestational age accounted for most of the variance, between 20% and 22% (standardized coefficient β=0.491 (males); β=0.466 (females)). PSM effect size was small, but significant for females (F=6.38; η^2=0.001; p= .012).

S1 Table. Analysis of the variance of the final models

Males	DF	Sums of Squares	F	η^2
Model	11	253472447.21	152.740[a]	0.361
Maternal age	1	1508018.47	9.996[a]	0.002
Weight gain during pregnancy	1	22614399.29	149.899[a]	0.032
Pre-pregnancy BMI	1	20989856.95	139.131[a]	0.030
Gestational age at delivery	1	157884469.63	1046.533[a]	0.225
PSM	1	543082.85	3.600[b]	0.001
HDP	1	939515.30	6.228[a]	0.001
GDM	1	872532.18	5.784[a]	0.001
Parity	1	21391981.87	141.796[a]	0.030
Smoking status	3	13664691.49	30.192[a]	0.019
Females	DF	Sums of Squares	F	η^2
Model	9	216110337.09	174.480[a]	0.367
Gestational age at delivery	1	121859184.35	885.465[a]	0.207
Weight gain during pregnancy	1	23643757.78	171.803[a]	0.040
Pre-pregnancy BMI	1	27036396.91	196.454[a]	0.046
PSM	1	878300.71	6.382[a]	0.001
HDP	1	2052243.58	14.912[a]	0.003
Parity	1	17621231.76	128.041[a]	0.030
Smoking status	3	7889158.68	19.108[a]	0.013

DF: degrees of freedom; BMI: body mass index; PSM: Psychological stress measure; HDP: hypertensive disorders of pregnancy; GDM: gestational diabetes mellitus; [a]p<.05; [b]p=.06

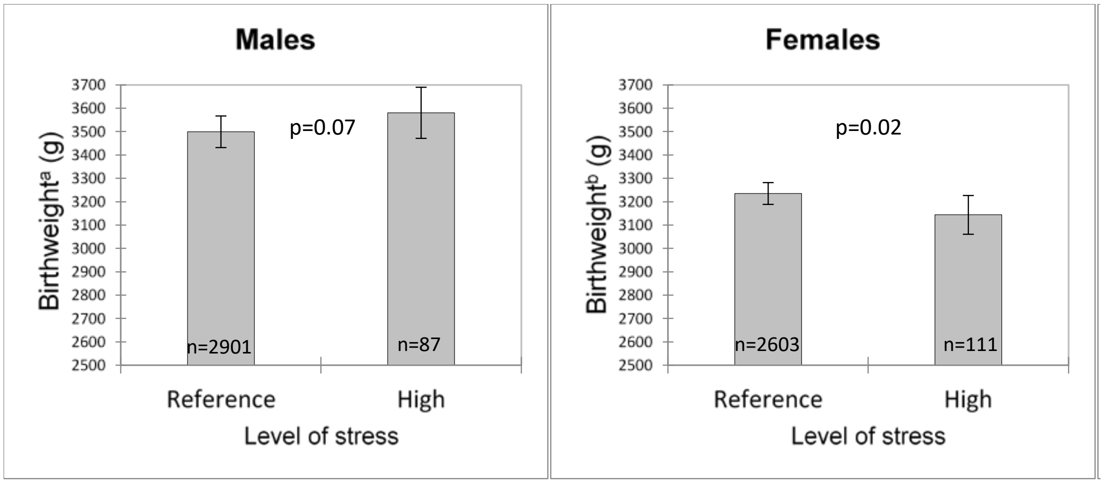

S1 Fig. Neonate birthweight as a function of maternal psychological stress during pregnancy

Results presented as Least Square (LS) means with $_{95\%}$CI; [a]adjusted for maternal age, pre-pregnancy BMI, weight gain during pregnancy, parity, presence of GDM and HDP, gestational age at delivery and smoking status; [b]adjusted for pre-pregnancy BMI, weight gain during pregnancy, parity, presence of HDP, gestational age at delivery and smoking status
