# Supplementary material for: Sex-specific association of high maternal psychological stress during pregnancy on newborn birthweight
Source: PLoS One. 2022 Jan 20;17(1):e0262641. doi: 10.1371/journal.pone.0262641 (PMC8775189; doi:10.1371/journal.pone.0262641)
Supplement: S2 File — (PDF) [file pone.0262641.s002.pdf]

Neonate birthweight Z-score as a function of maternal psychological stress during pregnancy: Analyses by sex of the neonate

To further measure whether female and male neonates differ within levels of stress, we used standardized sex-specific birthweight Z-scores for gestational age in analysis. Birthweight Z-scores were calculated using reference curves derived from population-based Canadian reference. S2 Fig shows the neonate birthweight Z-score as a function of newborn sex and maternal psychological stress levels during pregnancy. After adjustment for maternal age, pre-pregnancy BMI, weight gain during pregnancy, parity, presence of GDM and HDP, gestational age at delivery household income, and smoking status, very high PSM (>97.5th percentile) was significantly linked to increased birthweight Z-score by 0.215 ($CI_{95\%}$ [0.021 – 0.409]; p= .03) in males. Conversely, greater levels of PSM was linked to lower birthweight Z-score by -0.218 in females ($CI_{95\%}$ [-0.047 – -0.390]; p= .013) in a model adjusted for pre-pregnancy BMI, weight gain during pregnancy, gestational age at delivery and smoking status. PSM effect size was small, but significant for males and females (males: F=5.10; η^2=0.001; p= .024)(females: F=6.86; η^2=0.002; p= .009).

S2 Table. Analysis of the variance of the final models

Males	DF	Sums of Squares	F	η^2
Model	11	351.52	42.27[a]	0.135
Maternal age	1	8.67	11.46[a]	0.003
Weight gain during pregnancy	1	107.07	141.62[a]	0.041
Pre-pregnancy BMI	1	106.79	141.25[a]	0.041
Gestational age at delivery	1	15.05	19.90[a]	0.006
PSM	1	3.85	5.10[a]	0.001
HDP	1	4.88	6.45[a]	0.002
GDM	1	3.37	4.46[a]	0.001
Parity	1	95.89	126.83[a]	0.037
Smoking status	3	65.37	28.82[a]	0.025
Females	DF	Sums of Squares	F	η^2
Model	9	345.82	52.38[a]	0.148
Gestational age at delivery	1	4.14	5.64[a]	0.002
Weight gain during pregnancy	1	127.17	173.36[a]	0.055
Pre-pregnancy BMI	1	143.13	195.12[a]	0.061
PSM	1	5.04	6.86[a]	0.002
HDP	1	12.14	16.55[a]	0.005
Parity	1	81.81	111.52[a]	0.035
Smoking status	3	37.71	17.13[a]	0.016

DF: degrees of freedom; BMI: body mass index; PSM: Psychological stress measure; HDP: hypertensive disorders of pregnancy; GDM: gestational diabetes mellitus; [a]$p<.05$

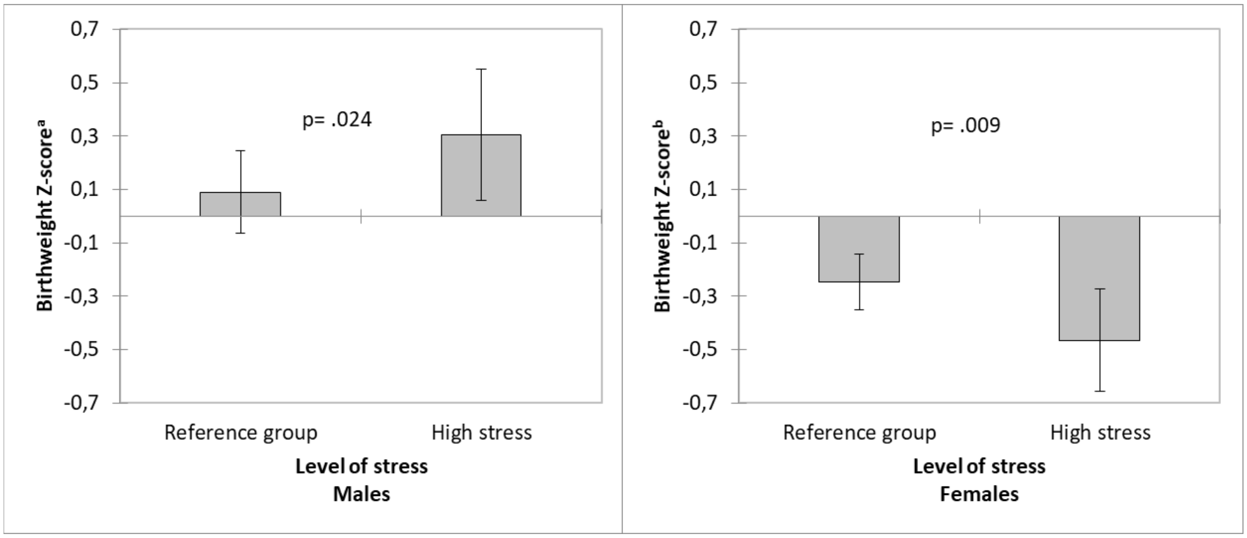

S2 Fig. Neonate birthweight Z-score as a function of sex of the newborn according to maternal psychological stress during pregnancy. Results presented as Least Square (LS) means with 95%CI; [a]adjusted for maternal age, pre-pregnancy BMI, weight gain during pregnancy, parity, presence of GDM and HDP, gestational age at delivery, and smoking status; [b]adjusted pre-pregnancy BMI, weight gain during pregnancy, gestational age at delivery, parity, presence of HDP and smoking status
