# Supplementary material for: Sex-specific association of high maternal psychological stress during pregnancy on newborn birthweight
Source: PLoS One. 2022 Jan 20;17(1):e0262641. doi: 10.1371/journal.pone.0262641 (PMC8775189; doi:10.1371/journal.pone.0262641)
Supplement: S3 File — (PDF) [file pone.0262641.s003.pdf]

Term neonate birthweight as a function of maternal psychological stress during pregnancy

Since birthweight was strongly related to gestational age, we also performed the analysis after excluding preterm births. There were no births prior to 30 weeks of gestation. 294 infants (5.16%) were born prior to 37 weeks (167 in males, 127 in females). When analyses were conducted solely in full-term births (≥37 wks), results were unchanged in both combined and independent stratified by neonate sex analyzes. In the combined model (males + females), 26% of birthweight variance was explained by the variables in the model: maternal age, pre-pregnancy BMI, weight gain during pregnancy, parity, presence of GDM and HDP, gestational age at delivery, smoking status, level of stress, sex of newborn, and the stress by newborn sex interaction. The stress by newborn interaction term was statistically significant, accounting for 0.1% of birthweight variance (η^2 0.0012; p= .003). Comparisons revealed significant differences in birthweight: There was a trend for male newborns whose mother experienced high levels of PSM to have greater birthweight (Difference: 73.16 g $CI_{95\%}$ [-17.46 – 163.79]; p= .11), whereas female newborns in the same circumstances had significantly lower birthweight (Difference: -94.49 g $CI_{95\%}$ [-17.81 – -171.18]; p= .016).

Analyses performed independently for male and female infants at term supported these observations (males: 75.57 g $CI_{95\%}$ [-15.71 – 166.86]; p= .11)(Females: -97.79 g $CI_{95\%}$ [-20.78 – -174.80]; p= .013).

S3 Table. Analysis of the variance of the final models

Males + Females	DF	Sums of Squares	F	η^2
Model	13	278333353.48	147.84[a]	0.26270
Maternal age	1	1697039.14	11.72[a]	0.00160
Weight gain during pregnancy	1	120309420.54	830.72[a]	0.11355
Pre-pregnancy BMI	1	42604905.56	294.18[a]	0.04021
Gestational age at delivery	1	43074595.17	297.42[a]	0.04065
PSM	1	20016.42	0.138[a]	0.00002
HDP	1	1677074.74	11.58[a]	0.00158
GDM	1	1332445.87	9.20[a]	0.00126
Parity	1	36553362.97	252.40[a]	0.03450
Sex of the newborn	1	7212074.23	49.80[a]	0.00681
Smoking status	3	20263694.04	46.64[a]	0.01913
Level of stress*sex of newborn	1	1240303.67	8.56[a]	0.00117
Males	DF	Sums of Squares	F	η^2
Model	11	136373062.82	82.22[a]	0.24356
Maternal age	1	1611977.19	10.69[a]	0.00288
Weight gain during pregnancy	1	67422313.31	447.16[a]	0.12042
Pre-pregnancy BMI	1	21108242.76	139.99[a]	0.03770
Gestational age at delivery	1	19311168.78	128.08[a]	0.03449
PSM	1	449384.53	2.98	0.00080
HDP	1	508433.02	3.37	0.00091
GDM	1	890313.45	5.91[a]	0.00159
Parity	1	20737121.91	137.53[a]	0.03704
Smoking status	3	12590770.75	27.84[a]	0.02249
Females	DF	Sums of Squares	F	η^2
Model	9	121419208.27	97.29[a]	0.25360
Gestational age at delivery	1	51868383.84	374.04[a]	0.10834
Weight gain during pregnancy	1	21819000.37	157.34[a]	0.04557
Pre-pregnancy BMI	1	25669683.84	185.11[a]	0.05362
PSM	1	946950.23	6.83[a]	0.00198
HDP	1	1257842.39	9.07[a]	0.00263
Parity	1	16261037.15	117.26[a]	0.03396
Smoking status	3	7248286.40	17.42[a]	0.01514

DF: degrees of freedom; BMI: body mass index; PSM: Psychological stress measure; HDP: hypertensive disorders of pregnancy; GDM: gestational diabetes mellitus; [a]p<.05

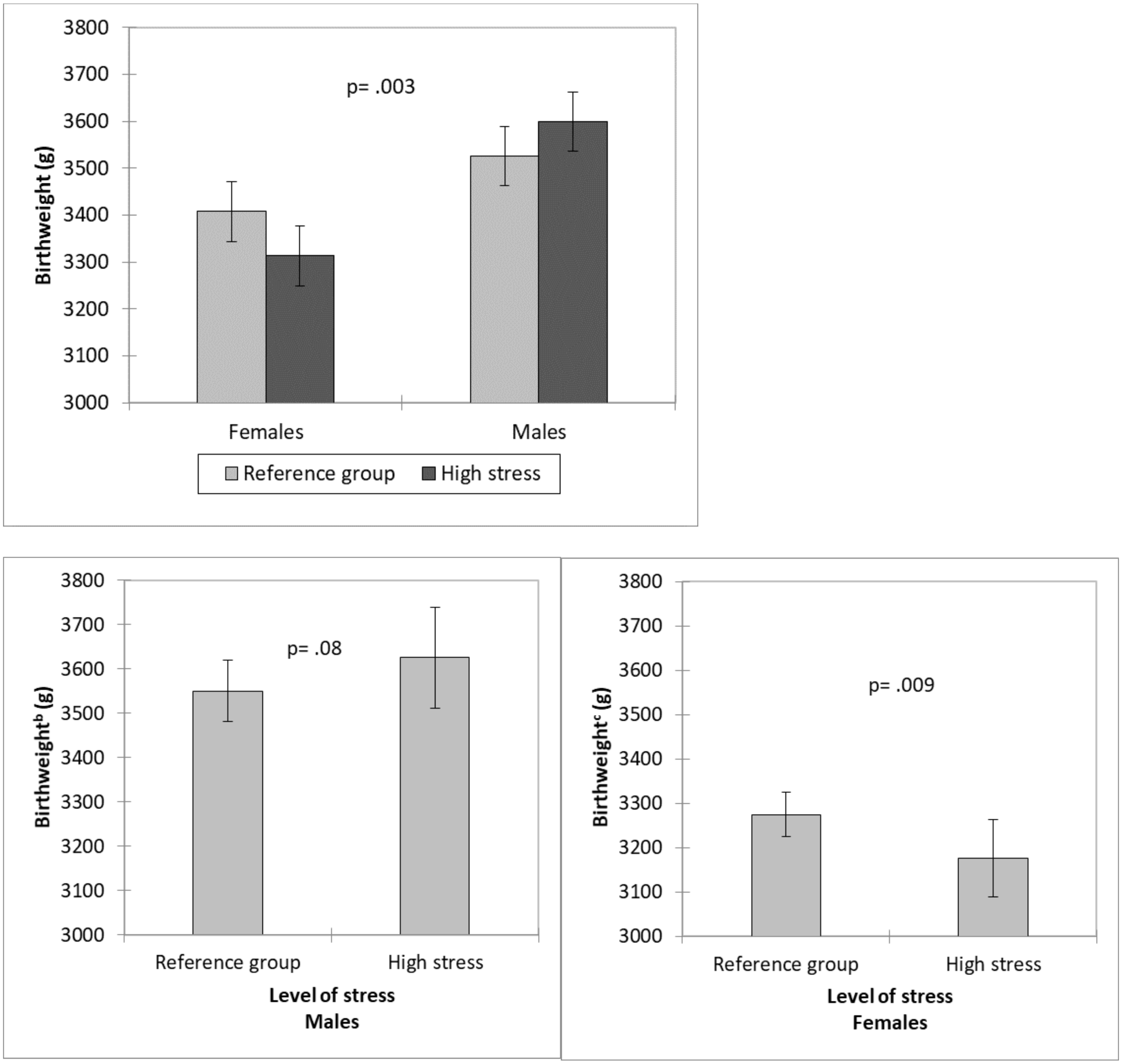

S3 Fig. Term neonate birthweight as a function of maternal psychological stress during pregnancy and newborn sex. Results presented as Least Square (LS) means with $CI_{95\%}$; [a]variables included in the model: maternal age, pre-pregnancy BMI, weight gain during pregnancy, parity, presence of GDM and HDP, gestational age at delivery, smoking status, level of stress, sex of the newborn and 'level of stress*sex of the newborn' interaction;

[b]variables included in the model: maternal age, pre-pregnancy BMI, weight gain during pregnancy, parity, presence of GDM and HDP, gestational age at delivery, smoking status, level of stress;

[c]variables included in the model: pre-pregnancy BMI, weight gain during pregnancy, parity, presence of HDP, gestational age at delivery, smoking status, level of stress
